# Association of Hypoxia-Induced Lactate Accumulation with AARS2 Expression, PDHA1/CPT2 Lactylation, and Energy Metabolism in Yak Skeletal Muscle Cells

**DOI:** 10.3390/cells15171515

**Published:** 2026-08-23

**Authors:** Tianshuai Li, Zhengbo Li, Jialin Wang, Miaoran Li, Kangfei An, Qixin Wang, Guoxiu Li, Lingxia Li, Jianshu Lv, Xiangdong Ye, Xiaodong Ling

**Affiliations:** 1 College of Agriculture and Animal Husbandry, Qinghai University, Xining 810016, China; lts431893827@163.com (T.L.); 18434898840@163.com (J.W.); limiaoran0416@163.com (M.L.); 13783721750@163.com (K.A.); 19963826401@163.com (Q.W.); liguoxiu1122@163.com (G.L.); lilingxia852@126.com (L.L.); ivjs153@163.com (J.L.); 2 Gaomiao Town Animal Husbandry and Veterinary Station, Ledu District Bureau of Agriculture and Rural Affairs, Haidong 810799, China

**Keywords:** alanyl-transfer RNA synthetase 2, lactylation, energy metabolism, hypoxic response, yak

## Abstract

**Highlights:**

**What are the main findings?**

- AARS2 expression was generally higher, whereas PDHA1 and CPT2 expression was lower, in oxygen-sensitive tissues from yaks in the high-altitude sampling group than in the lower-altitude groups. Galloflavin treatment reduced intracellular lactate levels and was accompanied by decreased AARS2 and increased PDHA1 and CPT2 protein expression in yak skeletal muscle cells.
- Hypoxia increased intracellular lactate levels and global protein lactylation in yak skeletal muscle cells. PDHA1 and CPT2 were identified as lactylated proteins, and AARS2 knockdown increased their protein expression. These molecular changes were associated with increased glycolysis-related indicators and decreased mitochondrial oxidative phosphorylation-related indicators under hypoxic conditions.

**What are the implications of the main findings?**

- These findings provide new insights into the associations among lactate, AARS2 expression, PDHA1/CPT2 lactylation, and hypoxia-related metabolic remodeling in yak skeletal muscle.

**Abstract:**

Yaks (*Bos grunniens*) are a typical species inhabiting high-altitude environments; however, their molecular responses to hypoxic conditions remain incompletely understood. This study aimed to investigate associations among alanyl-transfer RNA synthetase 2 (AARS2) expression, lactylation of pyruvate dehydrogenase E1 alpha 1 (PDHA1) and carnitine palmitoyltransferase 2 (CPT2), and energy metabolism under hypoxic conditions in yaks. Oxygen-sensitive tissues (skeletal muscle, cardiac muscle, and lungs) collected from yaks at different altitudes, along with primary yak skeletal muscle cells, were analyzed using RT-qPCR, Western blotting, immunofluorescence (IF), and co-immunoprecipitation (co-IP). In tissues from the three altitude groups, AARS2 expression was higher in the higher-altitude groups (*p* < 0.05), whereas PDHA1 and CPT2 expression was lower (*p* < 0.01). Overall protein lactylation levels were also higher in tissues from the higher-altitude groups. Under hypoxic conditions, lactate levels and AARS2 expression increased in skeletal muscle cells, accompanied by increased activities of key glycolytic enzymes and decreased activities of enzymes related to mitochondrial oxidative phosphorylation. Galloflavin treatment reduced lactate levels and partially attenuated these metabolic changes. Endogenous co-IP detected lactylation of both PDHA1 and CPT2. Following siRNA-mediated knockdown of AARS2, the protein abundance of PDHA1 and CPT2 increased. Taken together, these findings support an association between hypoxia-induced lactate accumulation, increased AARS2 expression, PDHA1/CPT2 lactylation, and a shift in cellular energy metabolism toward glycolysis. This study provides a theoretical basis for further understanding hypoxia-related metabolic remodeling in yaks.

## 1. Introduction

The Qinghai–Tibet Plateau is characterized by hypoxia, extreme cold, and intense radiation, with hypoxia as the most distinctive feature and a key ecological factor limiting animal survival [1,2,3]. As a keystone species of the plateau ecosystem [4], the yak (*Bos grunniens*) has significant ecological and economic value and is widely used as a model for investigating physiological and molecular characteristics associated with high-altitude environments [5]. However, current research on high-altitude adaptation in yaks has largely focused on genomic evolution, physiological phenotypes, and traditional transcriptional regulatory pathways [6,7]; little is known about post-translational regulatory changes associated with hypoxic responses. In recent years, protein lactylation, a newly identified acyl post-translational modification, has been shown to contribute to cellular responses to hypoxic stress by regulating metabolic enzyme activity and cellular energy metabolism [8], providing a new perspective for investigating metabolic responses to hypoxia in plateau animals.

Protein lysine lactylation was first identified by Zhang et al. [9] as a histone modification in macrophages. Histone lactylation levels are regulated by intracellular L-lactate concentrations and can influence downstream gene transcription [10]. Previous studies have shown that lactylation is not limited to histones but also occurs widely in non-histone proteins [11], and may regulate cellular metabolism by influencing protein conformation, molecular interactions, and enzyme activity [12]. Under hypoxic conditions, intracellular lactate levels can increase and are associated with enhanced lactylation [13]. Mao et al. [14] reported that AARS2 possesses lactyltransferase activity and catalyzes the lactylation of PDHA1 and CPT2, which are key enzymes in mitochondrial metabolism, thereby reducing their activity, impairing mitochondrial oxidative phosphorylation, and promoting a shift in cellular energy metabolism toward glycolysis. Furthermore, AARS2 has been reported to function as an intracellular L-lactate sensor and to regulate various cellular functions by catalyzing global lysine lactylation [15].

Mitochondrial oxidative phosphorylation is a major pathway for adenosine triphosphate (ATP) synthesis in mammalian cells under aerobic conditions [16]. During this process, the pyruvate dehydrogenase complex (PDC) converts pyruvate into acetyl-CoA, whereas the carnitine palmitoyltransferase (CPT1/CPT2) system facilitates the mitochondrial transport and oxidation of long-chain fatty acids, thereby supplying substrates for the tricarboxylic acid (TCA) cycle [17,18]. As a core component of the PDC E1 enzyme, PDHA1 participates in pyruvate conversion to acetyl-CoA [19], while CPT2 plays an essential role in mitochondrial fatty acid oxidation by converting acylcarnitines back into acyl-CoA within the mitochondrial matrix [20]. Under hypoxic conditions, cells often reduce oxidative phosphorylation, enhance glycolysis to maintain ATP homeostasis, and reduce oxygen consumption [21,22]. Recent studies have shown that protein lactylation under hypoxic conditions may contribute to the regulation of intracellular homeostasis through changes in energy metabolism. Mao et al. [14] reported that, in their experimental models, hypoxia-induced lactate accumulation promoted AARS2-mediated lactylation of PDHA1 and CPT2, thereby reducing their enzymatic activities and decreasing the oxidative utilization of glucose and fatty acids, thereby lowering oxidative phosphorylation levels. These findings suggest that AARS2 may represent a regulatory link among hypoxia, lactate signaling, and energy metabolism. However, these findings were obtained primarily from in vitro cell lines and mouse models. Whether similar associations among hypoxia, protein lactylation, and energy metabolism occur in yak tissues and cells remains unclear. Therefore, investigating the association between protein lactylation and hypoxia-related metabolic responses in yaks is important.

In this study, we investigated associations among AARS2 expression, PDHA1/CPT2 lactylation, and energy metabolism in yak skeletal muscle cells to improve understanding of hypoxia-related metabolic responses in yaks. These findings provide preliminary information for further investigation of lactylation-associated metabolic regulation under hypoxic conditions.

## 2. Materials and Methods

### 2.1. Animals

All animal handling and sampling procedures were approved by the Animal Welfare and Research Ethics Committee of Qinghai University (IACUC Approval No. SL-2023005, 15 March 2023), and all procedures were conducted in accordance with the 3Rs principles for animal experimentation.

Nine healthy 12-month-old male yaks were included, with three animals sampled from each of three geographic regions at different altitudes in Qinghai Province: Guide County (low-altitude group, approximately 2200 m), Haiyan County (medium-altitude group, approximately 3200 m), and Golok County (high-altitude group, approximately 4200 m) (*n* = 3 per group; *n* = 9 in total). The geographic coordinates, exact altitude, climatic conditions, diet composition, housing environment, and routine management practices for each sampling region are summarized in Supplementary Table S1. The animal-tissue component of this study was designed as an exploratory cross-sectional comparison of yaks from three geographic regions at different altitudes. For the animal-tissue analyses, each individual yak constituted one independent biological replicate and was defined as the experimental unit. All animals were humanely slaughtered at licensed abattoirs following a standardized slaughter and tissue-sampling procedure in accordance with local livestock welfare standards, and skeletal muscle, cardiac muscle, and lung tissues were immediately harvested post-mortem. The interval from slaughter to tissue snap-freezing was maintained within 30 min for all animals. Tissue aliquots were fixed in 4% neutral buffered formalin for immunohistochemical analysis, and the remaining tissues were snap-frozen in liquid nitrogen and stored at −80 °C until RNA and protein extraction.

### 2.2. Cell Culture

Primary yak skeletal muscle cells were independently isolated from three donor animals, and cells from each animal were maintained separately throughout the experiments (*n* = 3 biological replicates). Cells derived from each donor animal constituted one biological replicate and one experimental unit. For each treatment condition, cells from each donor animal were cultured and analyzed independently. Replicate culture dishes from the same donor animal were considered technical replicates and were not treated as independent experimental units, and their measurements were averaged to generate a single value per animal before statistical analysis. Western blotting, ELISA, immunofluorescence, and co-immunoprecipitation were performed using three donor-derived biological replicates, with three technical replicates per biological replicate where applicable. For the biochemical assays of lactate, enzyme activities, and ATP described in Section 2.8, four independent donor-derived biological replicates were used. Cells were cultured in high-glucose DMEM (BL304A, Labgic, Shanghai, China) supplemented with 10% fetal bovine serum (Gibco, Grand Island, NY, USA) and 1% penicillin/streptomycin (G4003-100ML, Servicebio, Wuhan, China) at 37 °C in a humidified atmosphere containing 5% CO_2_ (Model 300557139, Thermo Fisher Scientific, Waltham, MA, USA). Upon reaching approximately 60% confluence, cells from each donor animal were assigned to two treatment conditions: normoxia (21% O_2_) in a standard CO_2_ incubator and hypoxia (3% O_2_) in a hypoxic incubator (Model 6731NK841276, Eppendorf AG, Hamburg, Germany) for 24 h.

### 2.3. RNA Extraction and RT-qPCR

Total RNA was extracted from approximately 50 mg of each tissue sample using TRIzol reagent. The extracted RNA was reverse-transcribed into cDNA using the FastKing cDNA First-Strand Synthesis Kit (KR116, TianGen, Beijing, China). Specific primers were designed based on the coding sequences of the yak *AARS2*, *PDHA1*, and *CPT2* genes obtained from NCBI (sequences shown in Table 1) and synthesized by Nanjing Anshengda Biotechnology Co., Ltd. RT-qPCR was performed using the FastFire Rapid Fluorescent Quantitative PCR Premix (FP207, TianGen, Beijing, China) on a real-time PCR system (FQD-96C, Bioer, Hangzhou, China). Each reaction was performed in a total volume of 20 µL, consisting of 10 µL of SYBR Green mixture, 1 µL of cDNA template, 0.6 µL each of forward and reverse primers, and 7.8 µL of ddH_2_O. The amplification program consisted of initial denaturation for 5 min, followed by 40 cycles of denaturation at 94 °C for 30 s, annealing at 60 °C for 30 s, and extension at 72 °C for 1 min. Each cDNA sample was analyzed in three technical replicates. Each altitude group comprised tissue samples from three independent animals (biological *n* = 3). The Ct values of the three technical replicates were averaged to obtain one value for each biological sample before statistical analysis. Relative gene expression was normalized to β-actin and calculated using the 2^−ΔΔ*Ct*^ method.

### 2.4. Western Blotting

Total protein was extracted from yak skeletal muscle, cardiac muscle, and lung tissues using an Animal Cell/Tissue Total Protein Extraction Kit (SD-001/SN-002; Inventbiotech, Beijing, China) according to the manufacturer’s instructions. Primary yak skeletal muscle cells were lysed in radioimmunoprecipitation assay (RIPA) buffer supplemented with a protease inhibitor cocktail. Protein concentrations were determined using a bicinchoninic acid (BCA) protein assay kit (CR2412054; Servicebio, Wuhan, China). Equal amounts of protein (30 µg per lane) were separated by sodium dodecyl sulfate–polyacrylamide gel electrophoresis using 5% stacking and 12% resolving gels and transferred onto polyvinylidene difluoride membranes (G6047-50-0.45; Servicebio). The membranes were blocked with 5% skim milk at 4 °C for 2 h and then incubated overnight at 4 °C with rabbit anti-AARS2, anti-PDHA1, and anti-CPT2 primary antibodies (stored in our laboratory; 1:1000), a polyclonal anti-lactate dehydrogenase antibody (SHBP0618; Bioprofile, Shanghai, China; 1:1000), or GAPDH (ZB15004-HRP-100; Servicebio; 1:2000). After washing with phosphate-buffered saline (PBS) with Tween 20, the membranes were incubated with goat anti-rabbit IgG secondary antibody (bs-40295G-IRDye800CW; Bioss, Beijing, China; 1:10,000) at room temperature for 2 h. Signals were developed using the BeyoECL Moon Ultra-Sensitive Chemiluminescence Kit (P0018FS; Beyotime, Shanghai, China) and an imaging system (Model 12003153, Bio-Rad Laboratories, Hercules, CA, USA), and the gray-scale values of the bands were quantified using ImageJ software(version 1.53a, National Institutes of Health, Bethesda, MD, USA).

### 2.5. IF

Primary yak skeletal muscle cells were seeded in confocal culture dishes and cultured under standard conditions until reaching an appropriate density, then subjected to normoxic or hypoxic treatment. After treatment, the culture medium was removed, and the cells were washed with PBS, fixed with 4% paraformaldehyde at room temperature for 20 min, and washed again with PBS. The cells were permeabilized with 0.1% Triton X-100 at room temperature for 10 min, then blocked with 5% BSA at room temperature for 30 min. Pan-Kla (1:1000) was added, and the cells were incubated overnight at 4 °C. After washing with PBS, a fluorescent secondary antibody (G1301-10ML; Servicebio) was added and the cells were incubated at room temperature in the dark for 1 h. Cell nuclei were counterstained with 4′,6-diamidino-2-phenylindole (DAPI) in the dark for 5 min, and then washed three times with PBS. Images were acquired using a laser-scanning confocal microscope to evaluate the relative intensity and cellular distribution of the Pan-Kla fluorescence signal.

### 2.6. Co-IP

A total of 500 μg of protein was extracted from yak skeletal muscle tissue, 4 μg of a PDHA1- or CPT2-specific antibody was added, and the mixture was incubated overnight at 4 °C on a rotary shaker. A total of 20 μL of Protein A/G agarose magnetic beads (G2237-50T, Servicebio, Wuhan, China) was added, and the mixture was incubated at 4 °C for 2 h. After centrifugation, the supernatant was discarded, and the magnetic beads were washed three times with pre-chilled PBS. A total of 40 μL of 2 × SDS loading buffer was added and boiled at 100 °C for 10 min, centrifuged, and the supernatant for Western blot analysis was collected. The primary antibody was Pan-Kla, and the secondary antibody was HRP-conjugated goat anti-rabbit IgG. GAPDH served as an internal control, and the membrane was developed using ECL. A Pan-Kla-positive band in the PDHA1 or CPT2 immunoprecipitation complexes confirmed that the protein was lactylated.

### 2.7. Galloflavin Treatment

Primary yak skeletal muscle cells from each donor animal were seeded separately in culture dishes and cultured under standard conditions until reaching approximately 60% confluence. The cells were divided into four groups: normoxia plus vehicle (N), normoxia plus galloflavin (Cat. No. HY-W040118, MedChemExpress LLC., Monmouth Junction, NJ, USA) (N+GF), hypoxia plus vehicle (H), and hypoxia plus galloflavin (H+GF). The N and N+GF groups were cultured in a standard incubator at 37 °C under normoxic conditions (21% O_2_ and 5% CO_2_), whereas the H and H+GF groups were cultured in a three-gas incubator at 37 °C under hypoxic conditions (3% O_2_, 5% CO_2_, and balance N_2_). The N+GF and H+GF groups were treated with galloflavin at a final concentration of 50 μM, whereas the N and H vehicle-control groups received an equal volume of dimethyl sulfoxide (DMSO), resulting in the same final DMSO concentration across all groups. After 24 h of treatment, the cells were washed three times with sterile PBS, lysed in RIPA lysis buffer for total protein extraction, and subjected to Western blotting as described above.

### 2.8. Measurement of Lactate, Enzyme Activities, and ATP

Cell lysates were collected from each experimental group and used for all subsequent biochemical assays. Intracellular lactate levels (MM-077001-96T), hexokinase (HK; MM-5100401-96T) and pyruvate kinase (PK; MM-5116501-96T) activities, succinate dehydrogenase (SDH; MM-5104601), mitochondrial respiratory chain complex I (MRCCI; MM-1019V1), and cytochrome c oxidase (COX; MM-1236V1) activities, as well as ATP levels (MM-9289701), were measured using the corresponding commercial assay kits. All assay kits were purchased from MEIMIAN (Jiangsu Enzyme-Immuno Biotechnology Co., Ltd., Yancheng, China). All assays were performed in accordance with the manufacturer’s instructions. For each treatment group, cells derived from four independent donor animals were analyzed as four biological replicates. Intracellular lactate and the levels of HK, PK, SDH, MRCCI, COX, and ATP were determined using the corresponding ELISA kits according to the manufacturer’s protocols. Absorbance values were measured using a microplate reader, and the levels of the corresponding analytes were calculated from their respective standard curves according to the manufacturer’s instructions. Quality-control procedures, including the appropriate blanks and standards, were performed in accordance with the manufacturer’s instructions for each assay.

### 2.9. AARS2 Small Interfering RNA (siRNA) Transfection

Two specific siRNAs targeting yak *AARS2* (XM_070374860.1) were designed and synthesized by GenePharma (Shanghai, China) (Table 1). Primary yak skeletal muscle cells were seeded into six-well plates. When cell confluence reached 60–70%, cells were transfected with AARS2 siRNA at a final concentration of 50 nM using Lipofectamine 3000 (Thermo Fisher Scientific, Carlsbad, CA, USA) according to the manufacturer’s instructions. A negative control group was included for comparison. Cells were harvested 48 h after transfection. Total protein was extracted, and AARS2 protein expression was assessed by Western blotting to verify knockdown efficiency. Changes in PDHA1 and CPT2 protein expression were measured to assess their expression following AARS2 knockdown. Each experimental group consisted of three biological replicates, each derived from an independent donor animal.

### 2.10. Statistical Analysis

Statistical analyses were performed using GraphPad Prism 9.0 (GraphPad Software, Inc., San Diego, CA, USA), with Microsoft Excel (Microsoft Corp., Redmond, WA, USA) used for data organization. All data are expressed as the mean ± standard error of the mean (SEM). For animal-tissue analyses, each individual yak was treated as one biological replicate and one experimental unit. For primary cell experiments, cells from each donor animal were treated as one biological replicate and one experimental unit, and technical replicates from the same donor were averaged before statistical analysis. Comparisons among the three independent altitude-associated sampling groups were performed using one-way analysis of variance (ANOVA). Comparisons between two donor-matched conditions in primary cell experiments were performed using a paired two-tailed Student’s *t*-test. Repeated-measures ANOVA was used for donor-matched primary cell experiments involving three or more conditions, as appropriate. Two-way repeated-measures ANOVA was used for donor-matched experiments involving two experimental factors. A value of *p* < 0.05 was considered statistically significant. In figures, *p* < 0.05, *p* < 0.01, *p* < 0.001, and *p* < 0.0001; ns indicates no significant difference.

## 3. Results

### 3.1. Expression Patterns of AARS2, PDHA1, and CPT2 in Yak Tissues Across Different Altitude Groups

**Figure 1:**
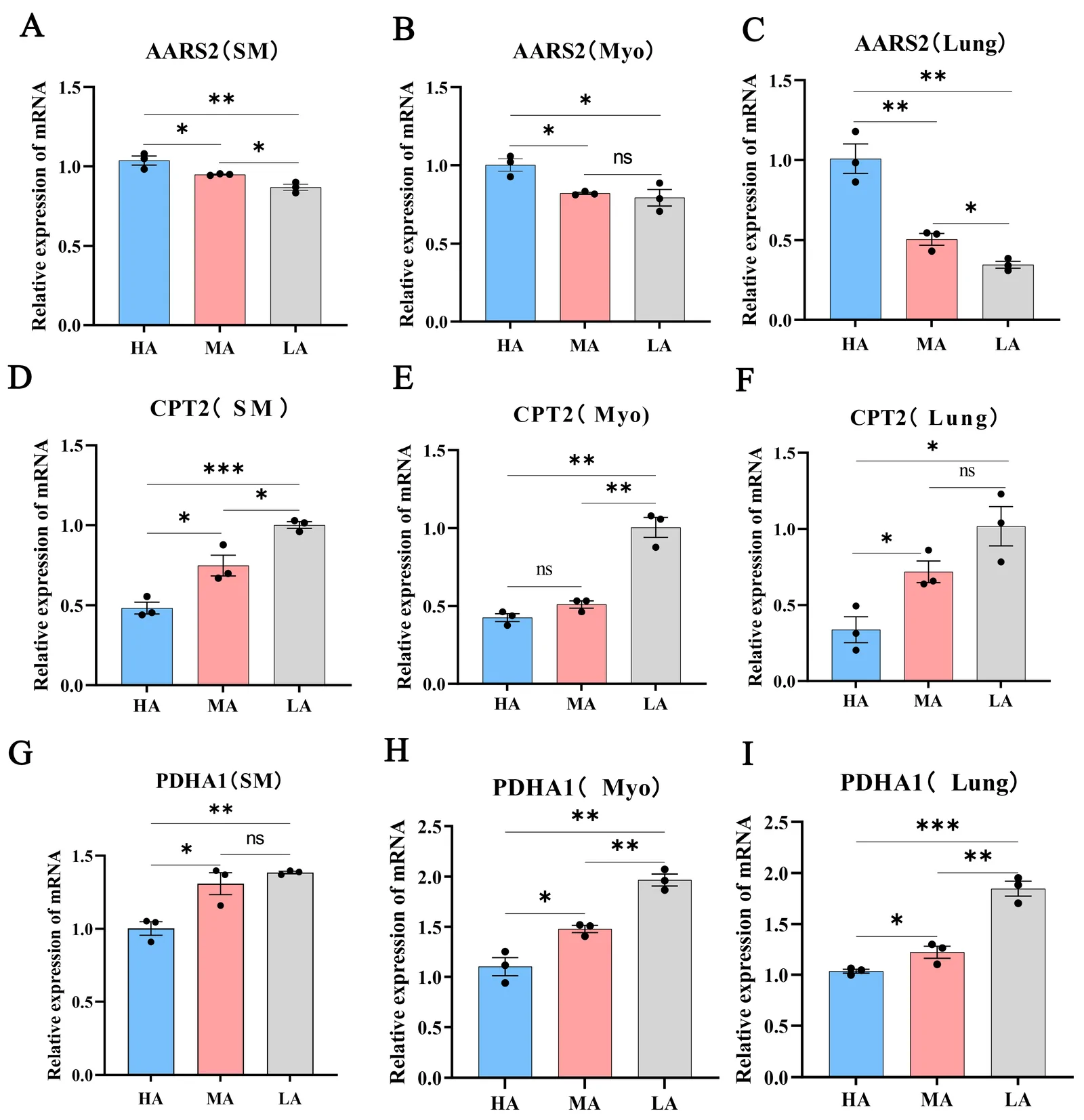
Expression of *AARS2*, *CPT2*, and *PDHA1* mRNA in yak tissues across different altitude groups. (**A**–**C**): Relative *AARS2* mRNA expression in skeletal muscle, cardiac muscle, and lung tissues from the high-altitude (HA), medium-altitude (MA), and low-altitude (LA) groups, respectively; (**D**–**F**): relative expression of CPT2 mRNA in the three tissues; (**G**–**I**): relative expression of *PDHA1* mRNA in the three tissues. β-actin served as the internal control. Data are presented as the mean ± SEM, with three animals per altitude group (*n* = 3 biological replicates). Individual data points represent independent animals. Statistical significance was assessed using one-way ANOVA followed by Tukey’s multiple-comparison test. * *p* < 0.05, ** *p* < 0.01, *** *p* < 0.001; ns, not significant. Exact *p* values for all pairwise comparisons are provided in Supplementary Table S2.

**Figure 2:**
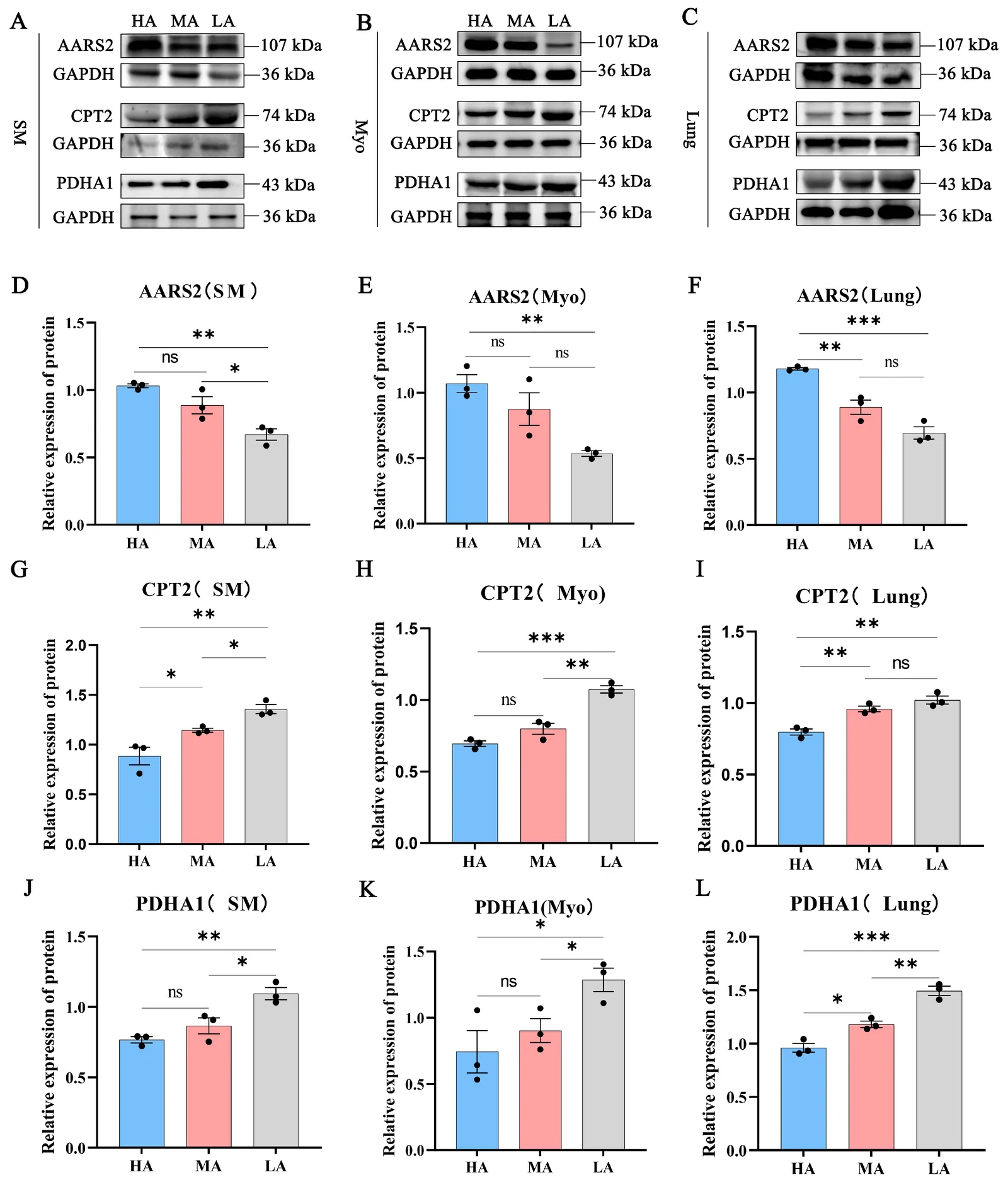
Protein expression levels of AARS2, CPT2, and PDHA1 in skeletal muscle, cardiac muscle, and lung tissues of yaks across different altitude groups. HA: high-altitude group; MA: medium-altitude group; LA: low-altitude group. (**A**–**C**): Representative Western blot images of AARS2, CPT2, and PDHA1 in skeletal muscle, cardiac muscle, and lung tissues, respectively; (**D**–**F**): relative AARS2 protein expression in skeletal muscle, cardiac muscle, and lung tissues; (**G**–**I**): relative CPT2 protein expression in skeletal muscle, cardiac muscle, and lung tissues; and (**J**–**L**): relative PDHA1 protein expression in skeletal muscle, cardiac muscle, and lung tissues. GAPDH served as the loading control. Data are presented as the mean ± SEM, with three animals per altitude group (*n* = 3 biological replicates). Individual data points represent independent animals. Statistical significance was assessed using one-way ANOVA followed by Tukey’s multiple-comparison test. * *p* < 0.05, ** *p* < 0.01, *** *p* < 0.001; ns, no significant difference. Exact *p* values for all pairwise comparisons are provided in Supplementary Table S2.

The mRNA and protein expression levels of *AARS2*, *PDHA1*, and *CPT2* were assessed in skeletal muscle, cardiac muscle, and lung tissues collected from yaks sampled from regions at low, medium, and high altitudes. Both the mRNA and protein expression levels of *AARS2* differed among the three sampling groups, with generally higher expression in the high-altitude group, particularly compared with the low-altitude group (Figure 1A–C and Figure 2A–F). In contrast, both the mRNA and protein expression levels of *CPT2* and *PDHA1* were generally lower in the high-altitude group, particularly compared with the low-altitude group, although the significance of individual pairwise comparisons varied among tissues (Figure 1D–I and Figure 2G–L). These findings showed a general expression pattern characterized by higher *AARS2* levels and lower *PDHA1* and *CPT2* levels in the high-altitude group. Exact *p* values for all pairwise comparisons are provided in Supplementary Table S2.

### 3.2. Hypoxia Increases AARS2 and Decreases PDHA1 and CPT2 Protein Expression in Yak Skeletal Muscle Cells

**Figure 3:**
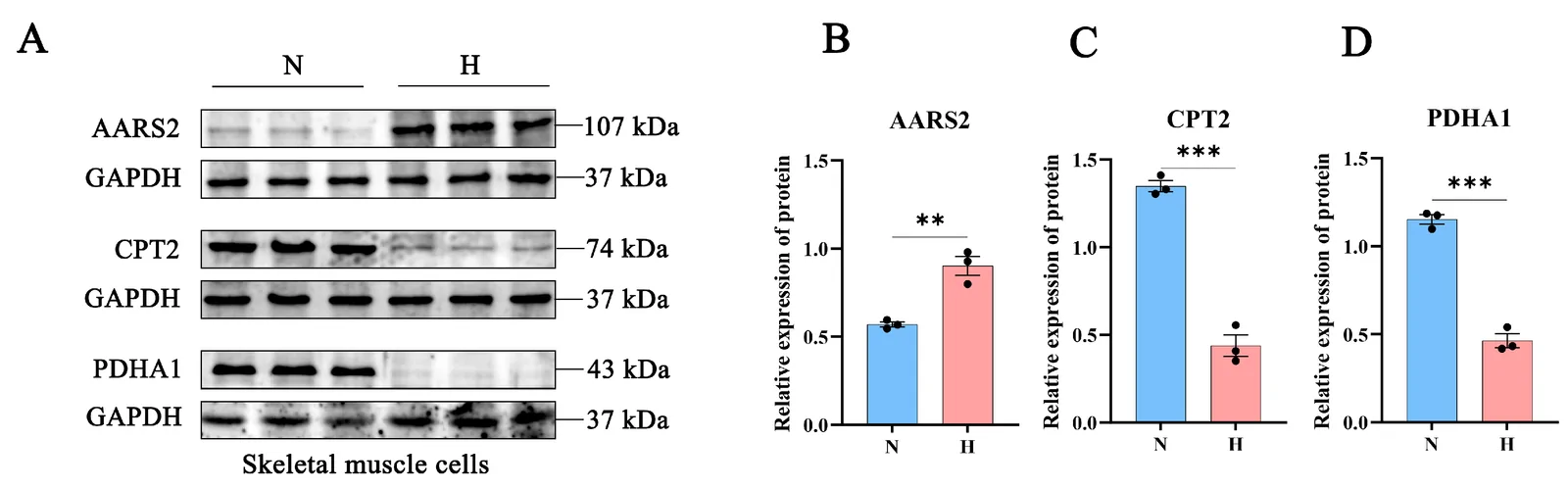
Protein expression of AARS2, PDHA1, and CPT2 in primary yak skeletal muscle cells under normoxic (N) and hypoxic (H) conditions. (**A**): Representative Western blot images for AARS2, CPT2, and PDHA1; (**B**–**D**): quantification of relative AARS2, CPT2, and PDHA1 protein expression, respectively. GAPDH served as the loading control. Data are presented as mean ± SEM from three independent donor-derived biological replicates (*n* = 3), with individual data points representing independent donor animals. Statistical significance was assessed using a paired two-tailed Student’s *t*-test. ** *p* < 0.01, *** *p* < 0.001.

To determine whether hypoxic exposure altered AARS2, PDHA1, and CPT2 protein expression, primary yak skeletal muscle cells were cultured under hypoxic conditions, and protein expression was analyzed by Western blotting. Compared with the normoxic group, AARS2 protein expression was significantly increased under hypoxic conditions (*p* = 0.0039), whereas CPT2 and PDHA1 protein expression levels were significantly decreased (CPT2, *p* = 0.0002; PDHA1, *p* = 0.0001) (Figure 3A–D). These cellular responses were broadly consistent with the expression patterns observed in yak tissue samples from the three altitude-associated sampling groups.

### 3.3. Hypoxia Increases Intracellular Lactate Levels and Global Protein Lactylation in Yak Skeletal Muscle Cells

**Figure 4:**
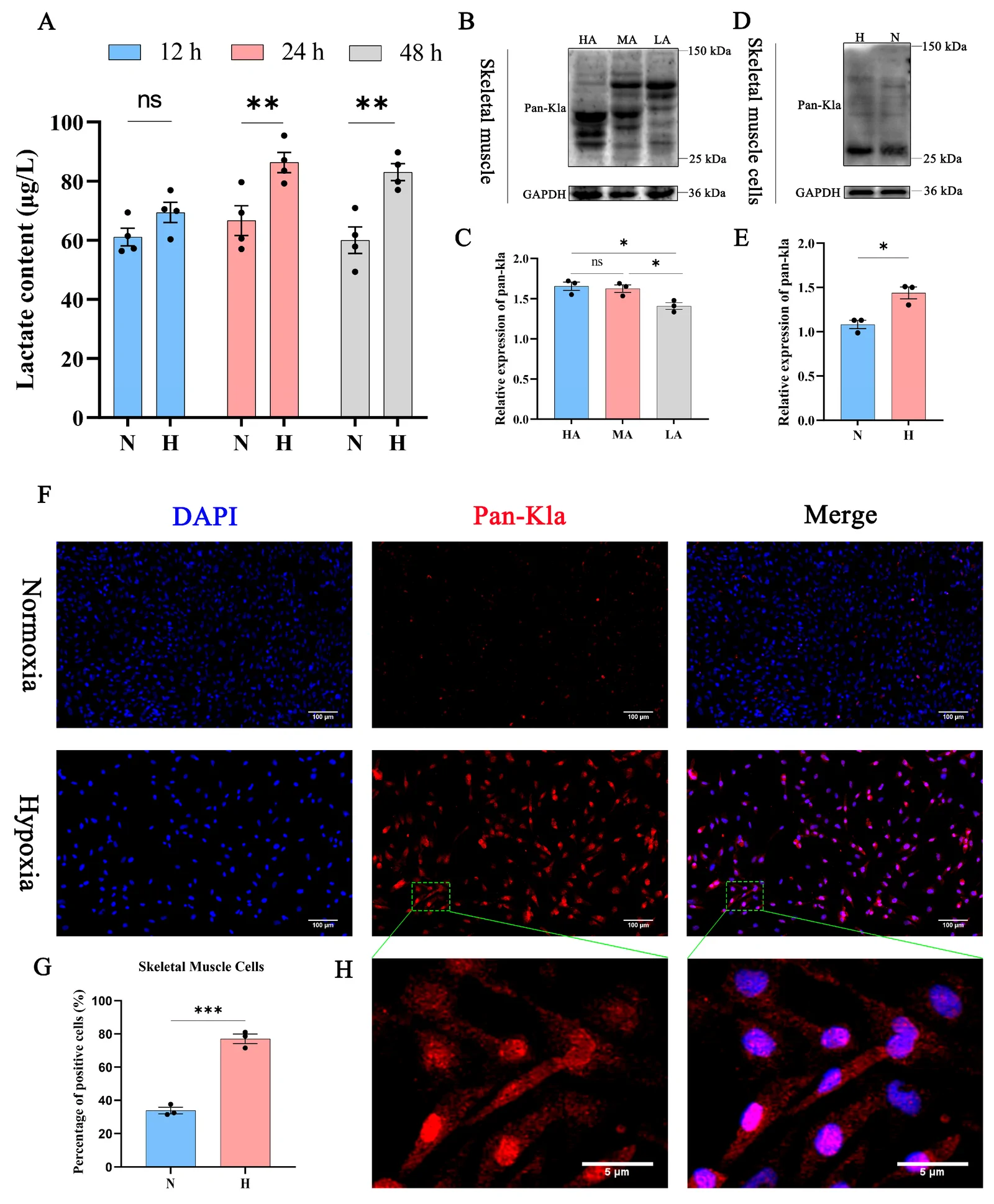
Intracellular lactate levels and global protein lactylation in yak skeletal muscle tissues and primary skeletal muscle cells. (**A**) Intracellular lactate levels in primary yak skeletal muscle cells under normoxic (N) and hypoxic (H) conditions after 12, 24, and 48 h of exposure. (**B**,**C**) Representative Western blot images and quantification of Pan-Kla levels in skeletal muscle tissues from the high-altitude (HA), medium-altitude (MA), and low-altitude (LA) sampling groups. (**D**,**E**) Representative Western blot images and quantification of Pan-Kla levels in primary yak skeletal muscle cells under normoxic and hypoxic conditions. (**F**–**H**) Representative Pan-Kla immunofluorescence images, quantification of Pan-Kla-positive cells, and magnified images of primary yak skeletal muscle cells under normoxic and hypoxic conditions. N, normoxia; H, hypoxia; HA, high-altitude group; MA, medium-altitude group; LA, low-altitude group. Data are presented as mean ± SEM. Tissue data represent three independent animals per sampling group (*n* = 3 biological replicates). Intracellular lactate data in panel A represent four independent donor-derived biological replicates (*n* = 4), whereas the other cell-based quantitative analyses in panels (**E**,**G**) represent three independent donor-derived biological replicates (*n* = 3). Individual data points represent independent animals or donor animals, as appropriate. Statistical significance was assessed using one-way ANOVA followed by Tukey’s multiple-comparison test for tissue comparisons (**C**), a paired two-tailed Student’s *t*-test for two-condition cell comparisons (**E**,**G**), and two-way repeated-measures ANOVA for the time-course experiment (**A**). * *p* < 0.05, ** *p* < 0.01, *** *p* < 0.001; ns, not significant.

Intracellular lactate levels in yak skeletal muscle cells under normoxic and hypoxic conditions were measured using enzyme-linked immunosorbent assay (ELISA). Compared with the normoxic group, intracellular lactate levels did not differ significantly after 12 h of hypoxic exposure (*p* = 0.3593), but were significantly higher in the hypoxic group at 24 h (*p* = 0.0052) and 48 h (*p* = 0.0013) (Figure 4A). Global protein lactylation levels in yak skeletal muscle tissues and cells were assessed using a Pan-Kla antibody. Pan-Kla levels in skeletal muscle tissue were significantly higher in the HA group than in the LA group (*p* = 0.0210) and were also higher in the MA group than in the LA group (*p* = 0.0255), whereas no significant difference was observed between the HA and MA groups (*p* = 0.6965) (Figure 4B,C). Similarly, Pan-Kla levels were significantly higher in hypoxia-treated skeletal muscle cells than in the normoxic group (*p* = 0.0124) (Figure 4D,E). Immunofluorescence analysis showed that Pan-Kla immunofluorescence signals were detectable in both the cytoplasm and nucleus of yak skeletal muscle cells. Compared with the normoxic group, Pan-Kla fluorescence was visibly enhanced under hypoxic conditions, and the percentage of Pan-Kla-positive cells was significantly higher in the hypoxic group than in the normoxic group (*p* = 0.0002) (Figure 4F–H). Collectively, these results showed that hypoxic exposure was accompanied by increased intracellular lactate levels and enhanced global protein lactylation in yak skeletal muscle cells.

### 3.4. Galloflavin Reduces Intracellular Lactate Levels and Is Accompanied by Changes in AARS2, CPT2, and PDHA1 Protein Expression Under Hypoxia

**Figure 5:**
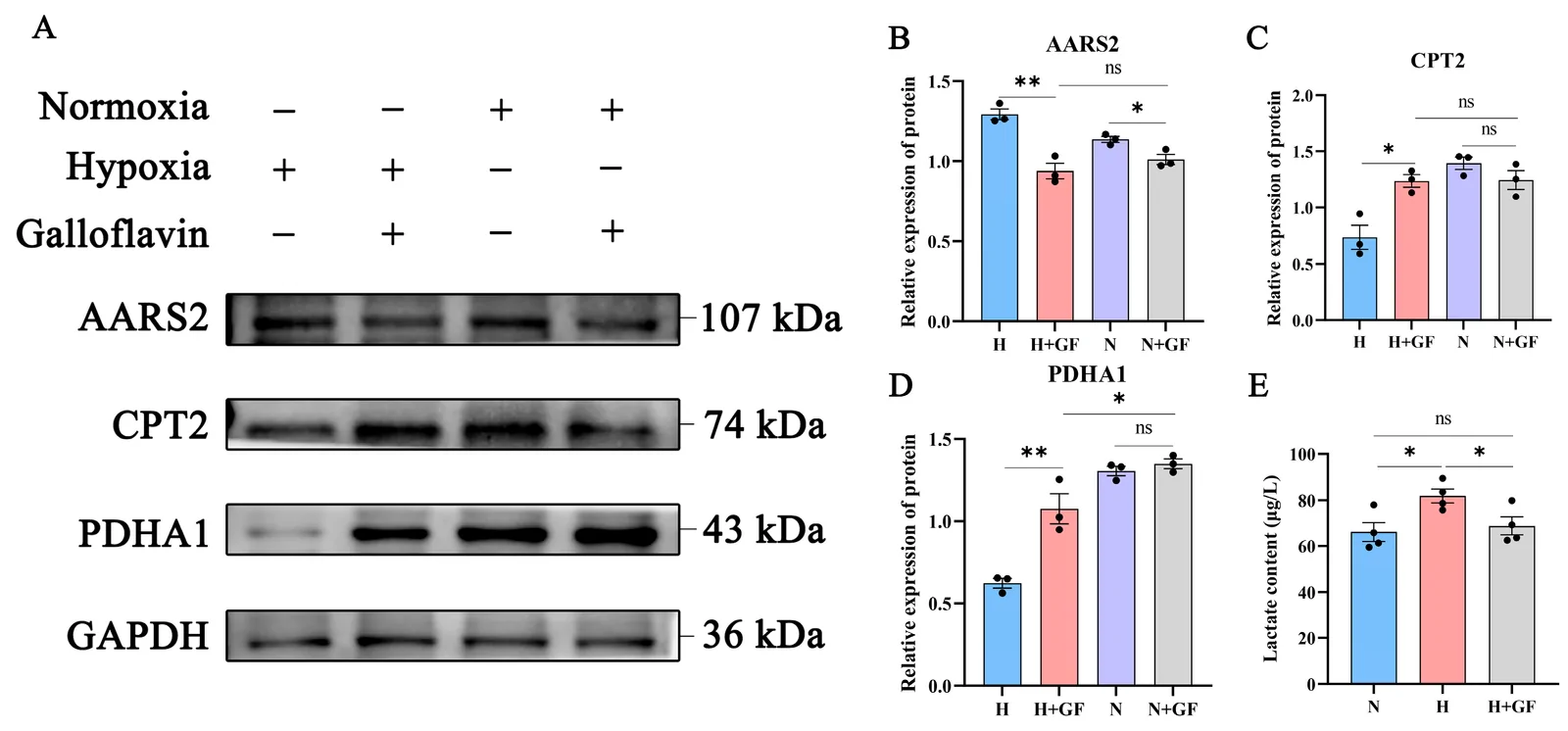
Effects of galloflavin treatment on intracellular lactate levels and AARS2, CPT2, and PDHA1 protein expression in primary yak skeletal muscle cells. (**A**) Representative Western blot images of AARS2, CPT2, and PDHA1 under normoxic, hypoxic, and galloflavin-treated conditions; GAPDH served as the loading control. (**B**–**D**) Quantification of relative protein expression of AARS2, CPT2, and PDHA1, respectively; (**E**) Intracellular lactate levels in the N, H, and H+GF groups. N, normoxia; N+GF, normoxia + galloflavin; H, hypoxia; H+GF, hypoxia + galloflavin. Data are presented as mean ± SEM. Protein-expression data in panels (**B**–**D**) represent three independent donor-derived biological replicates (*n* = 3), whereas intracellular lactate data in panel E represent four independent donor-derived biological replicates (*n* = 4). Individual data points represent independent donor animals. Statistical significance was assessed using two-way repeated-measures ANOVA for AARS2, CPT2, and PDHA1 protein expression (**B**–**D**) and one-way repeated-measures ANOVA for intracellular lactate levels (**E**), followed by appropriate multiple-comparison tests. * *p* < 0.05, ** *p* < 0.01; ns, not significant.

To examine whether changes in intracellular lactate levels were associated with changes in AARS2, CPT2, and PDHA1 expression, we used the lactate dehydrogenase inhibitor galloflavin to suppress lactate production, measured intracellular lactate levels via ELISA, and detected changes in the expression of AARS2, CPT2, and PDHA1 via Western blotting (Figure 5A). A preliminary CCK-8 assay was performed to evaluate the effects of different galloflavin concentrations on cell viability and to support the selection of the working concentration (Supplementary Figure S1A). At the selected concentration of 50 μM, galloflavin treatment for 24 h did not significantly reduce cell viability under either normoxic or hypoxic conditions compared with the corresponding DMSO vehicle controls (Supplementary Figure S1B). Intracellular lactate levels were significantly higher in the hypoxic group than in the normoxic group (*p* = 0.0221) and were significantly lower in the H+GF group than in the hypoxic group (*p* = 0.0396). No significant difference was observed between the H+GF and normoxic groups (*p* = 0.6556) (Figure 5E). In parallel, under hypoxic conditions, galloflavin treatment significantly decreased AARS2 protein expression (*p* = 0.0040) and increased CPT2 (*p* = 0.0140) and PDHA1 (*p* = 0.0093) protein expression compared with the untreated hypoxic group. Under normoxic conditions, galloflavin treatment also significantly decreased AARS2 protein expression (*p* = 0.0266), whereas no significant differences were observed in CPT2 (*p* = 0.2128) or PDHA1 (*p* = 0.3585) protein expression between the N and N+GF groups (Figure 5B–D). Collectively, galloflavin treatment under hypoxic conditions was accompanied by reduced intracellular lactate levels and partial attenuation of the hypoxia-associated changes in AARS2, CPT2, and PDHA1 protein expression.

### 3.5. Lactate Accumulation Is Associated with the Metabolic Shift from Oxidative Phosphorylation to Glycolysis in Hypoxic Yak Skeletal Muscle Cells

**Figure 6:**
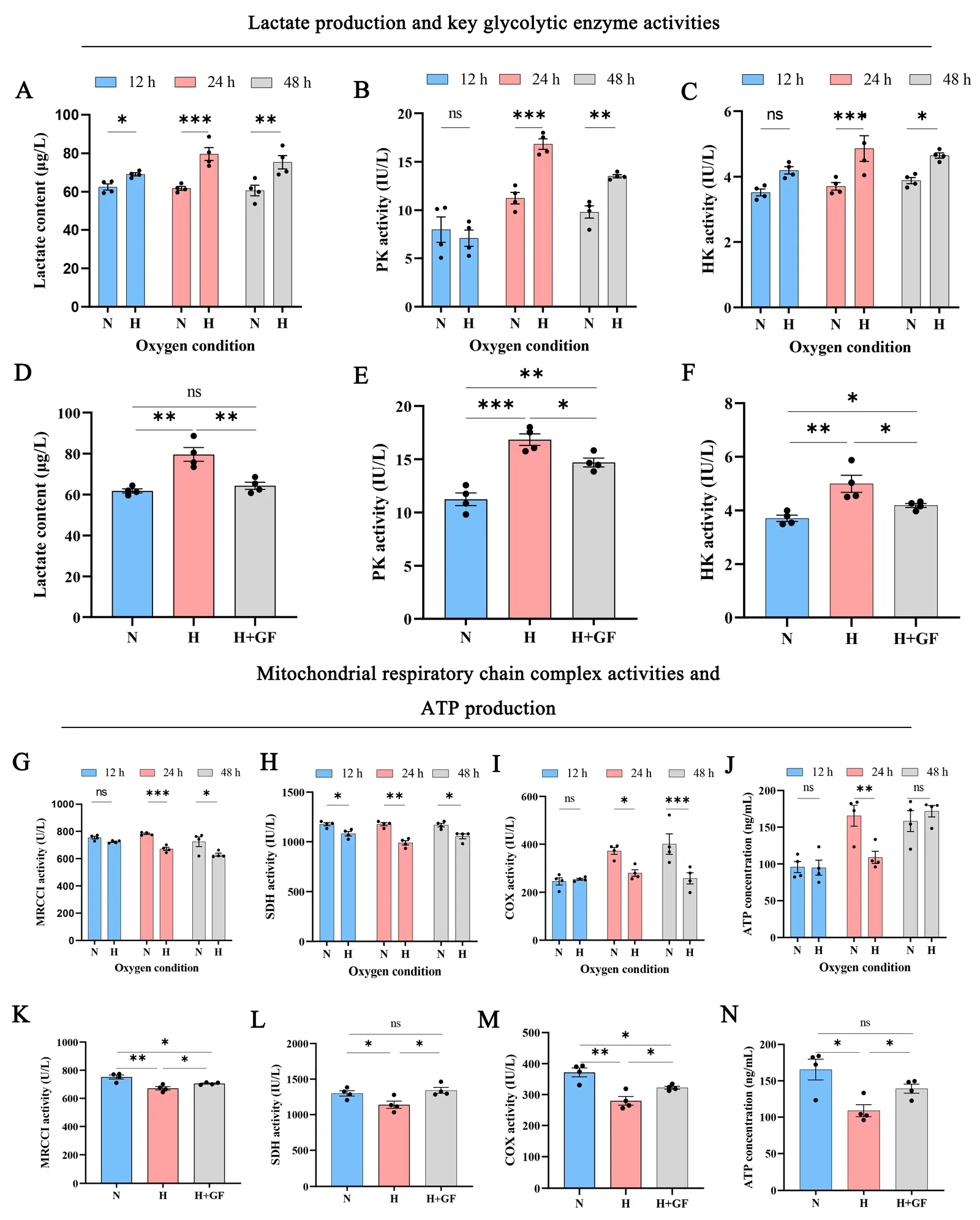
Effects of hypoxia and galloflavin treatment on glycolysis- and mitochondrial oxidative phosphorylation-related indicators in primary yak skeletal muscle cells. (**A**–**C**): Intracellular lactate levels and activities of pyruvate kinase (PK) and hexokinase (HK) in primary yak skeletal muscle cells after 12, 24, and 48 h of exposure to normoxic (N) and hypoxic (H) conditions; (**D**–**F**): Intracellular lactate levels, PK, and HK activities at 24 h in the N, H, and hypoxia + galloflavin (H+GF) groups; (**G**–**J**): mitochondrial respiratory chain complex I activity (MRCCI), succinate dehydrogenase (SDH) activity, and cytochrome c oxidase (COX) activity, and intracellular ATP levels after 12, 24, and 48 h of normoxic or hypoxic exposure; (**K**–**N**): MRCCI, SDH, and COX activities, and intracellular ATP levels in the N, H, and H+GF groups at 24 h. Data are presented as mean ± SEM from four independent donor-derived biological replicates (*n* = 4), with individual data points representing independent donor animals. Statistical significance was assessed using two-way repeated-measures ANOVA followed by Šídák’s mmultiple-comparisontest for the time-course experiments (**A**–**C**,**G**–**J**) and one-way repeated-measures ANOVA followed by Tukey’s multiple-comparisons test for the three-group comparisons (**D**–**F**,**K**–**N**). * *p* < 0.05, ** *p* < 0.01, *** *p* < 0.001; ns, not significant. Exact *p* values for all statistical comparisons are provided in Supplementary Table S3.

To clarify the effects of hypoxia on energy metabolism in yak skeletal muscle cells, energy metabolism-related indicators were measured via ELISA. Compared with the normoxic group, no significant differences in intracellular lactate levels or pyruvate kinase (PK) and hexokinase (HK) activities were observed after 12 h of hypoxic exposure, whereas these parameters were significantly increased at 24 and 48 h (Figure 6A–C; Supplementary Table S3). At 24 h, galloflavin treatment under hypoxic conditions significantly reduced intracellular lactate levels, accompanied by decreased PK and HK activities (Figure 6D–F; Supplementary Table S3). Analysis of mitochondrial oxidative phosphorylation-related parameters showed that hypoxia was associated with decreases in MRCCI, SDH, and COX activities and intracellular ATP levels, although the statistical significance of these changes varied among time points (Figure 6G–J; Supplementary Table S3). In contrast, galloflavin treatment under hypoxic conditions was accompanied by increased mitochondrial enzyme activities and intracellular ATP levels (Figure 6K–N; Supplementary Table S3). Taken together, these findings indicate that hypoxia-associated increases in intracellular lactate levels were accompanied by enhanced glycolysis-related indicators and reduced mitochondrial oxidative phosphorylation-related indicators, whereas galloflavin treatment partially attenuated these changes. These results support an association between lactate accumulation and hypoxia-related metabolic remodeling.

### 3.6. PDHA1 and CPT2 Undergo Lactylation in Yak Skeletal Muscle Cells

**Figure 7:**
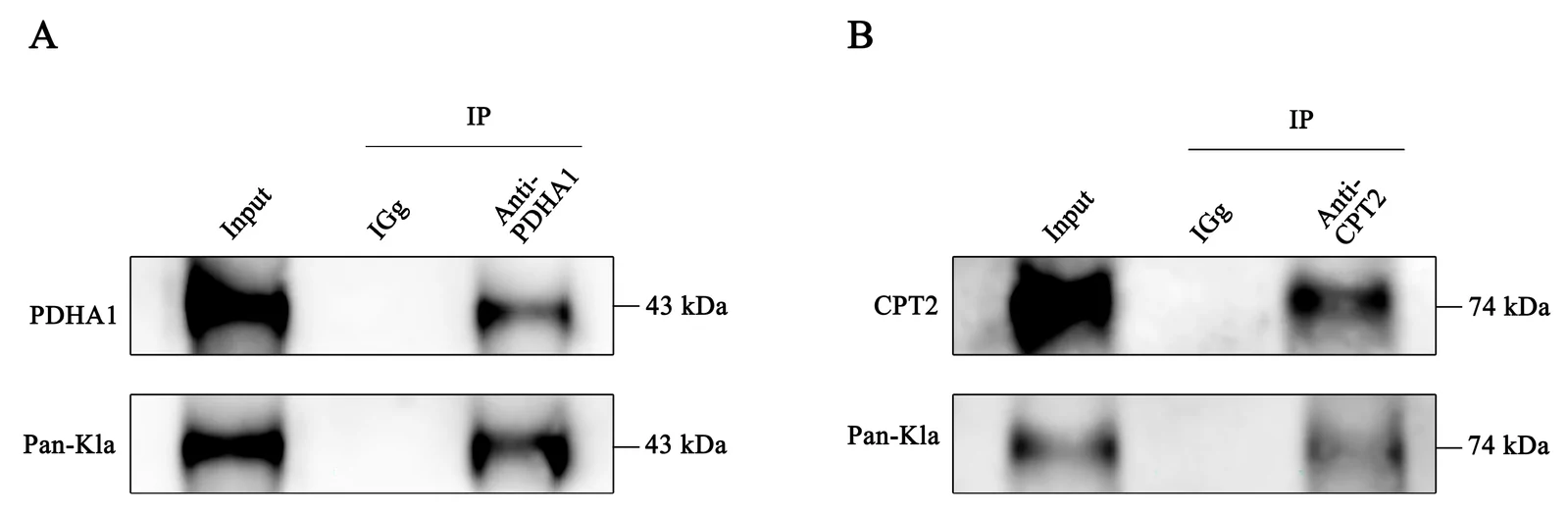
Identification of lysine lactylation modifications of PDHA1 and CPT2 in yak skeletal muscle cells by co-immunoprecipitation. (**A**): Immunoblotting of PDHA1 immunoprecipitates and (**B**): immunoblotting of CPT2 immunoprecipitates. Input, total cell lysate; IgG, negative control; anti-PDHA1 IP and anti-CPT2 IP, specific immunoprecipitation groups. Upper blots were probed with anti-PDHA1 or anti-CPT2 antibodies to confirm protein enrichment, and lower blots were probed with a Pan-Kla antibody to detect lactylation levels.

To determine whether PDHA1 and CPT2 undergo lactylation in yak skeletal muscle cells, we performed endogenous immunoprecipitation, followed by Western blotting. The immunoprecipitation (IP) results showed that both PDHA1 (43 kDa) and CPT2 (74 kDa) were specifically enriched by their respective antibodies. No specific bands were observed in the isotype IgG control group, whereas both target proteins were detected in the input group. Subsequent incubation with a Pan-Kla antibody resulted in the detection of specific bands in the immunoprecipitation products of both PDHA1 and CPT2, whereas no signal was observed in the IgG control group (Figure 7A,B). These results confirm that both PDHA1 and CPT2 undergo lactylation in yak skeletal muscle cells.

### 3.7. PDHA1 and CPT2 Protein Expression Increases Following AARS2 Knockdown

**Figure 8:**
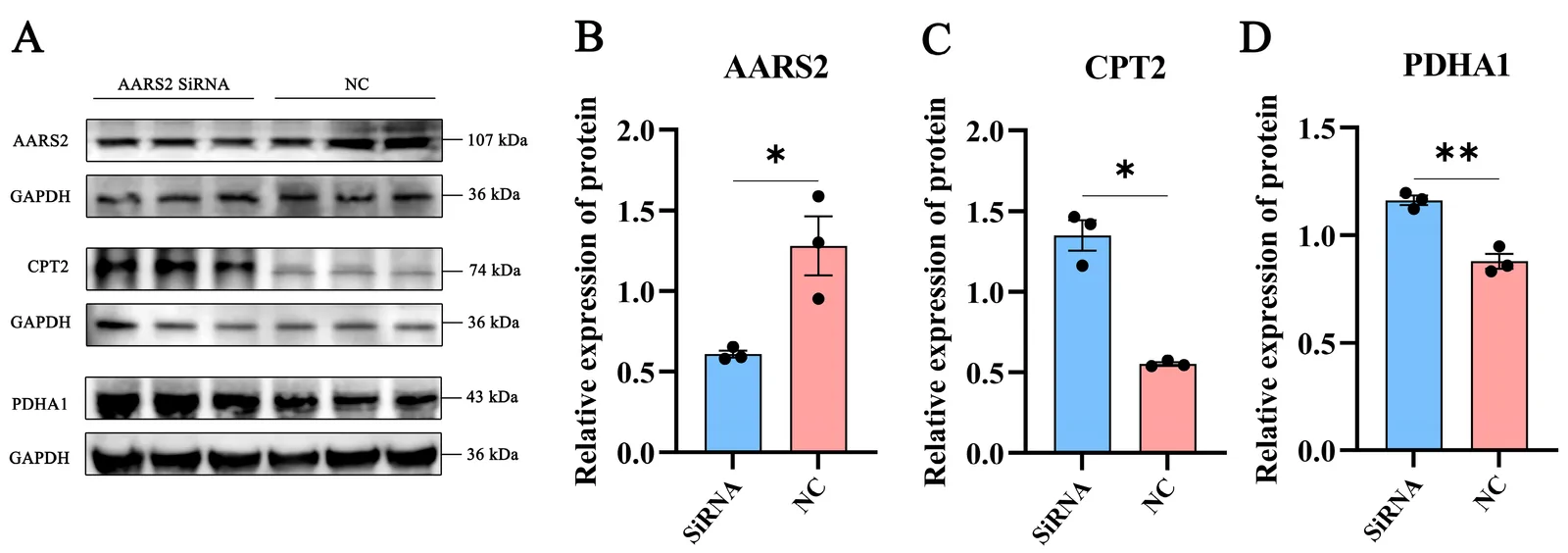
Effects of AARS2 silencing on protein expression of AARS2, CPT2, and PDHA1 in primary yak skeletal muscle cells. (**A**): Representative Western blot images of AARS2, CPT2, and PDHA1 in the AARS2 knockdown group (AARS2 siRNA) and the negative control group (NC); GAPDH served as the loading control. (**B**–**D**): Quantitative analysis of AARS2, CPT2, and PDHA1 protein bands. Data are presented as mean ± SEM from three independent donor-derived biological replicates (*n* = 3), with individual data points representing independent donor animals. Statistical significance was assessed using a paired two-tailed Student’s *t*-test. * *p* < 0.05, ** *p* < 0.01.

To investigate the relationship between AARS2 and CPT2 and PDHA1 protein expression, AARS2 was knocked down in yak skeletal muscle cells using AARS2-targeting siRNA. Western blot analysis showed that, compared with the negative control group, AARS2 protein was significantly downregulated in the AARS2 siRNA group (*p* = 0.0298) (Figure 8A,B), whereas CPT2 and PDHA1 protein expression levels were significantly increased (*p* = 0.0282 and *p* = 0.0024, respectively) (Figure 8C,D). These results showed that reduced AARS2 expression was accompanied by increased CPT2 and PDHA1 protein expression in yak skeletal muscle cells.

## 4. Discussion

**Figure 9:**
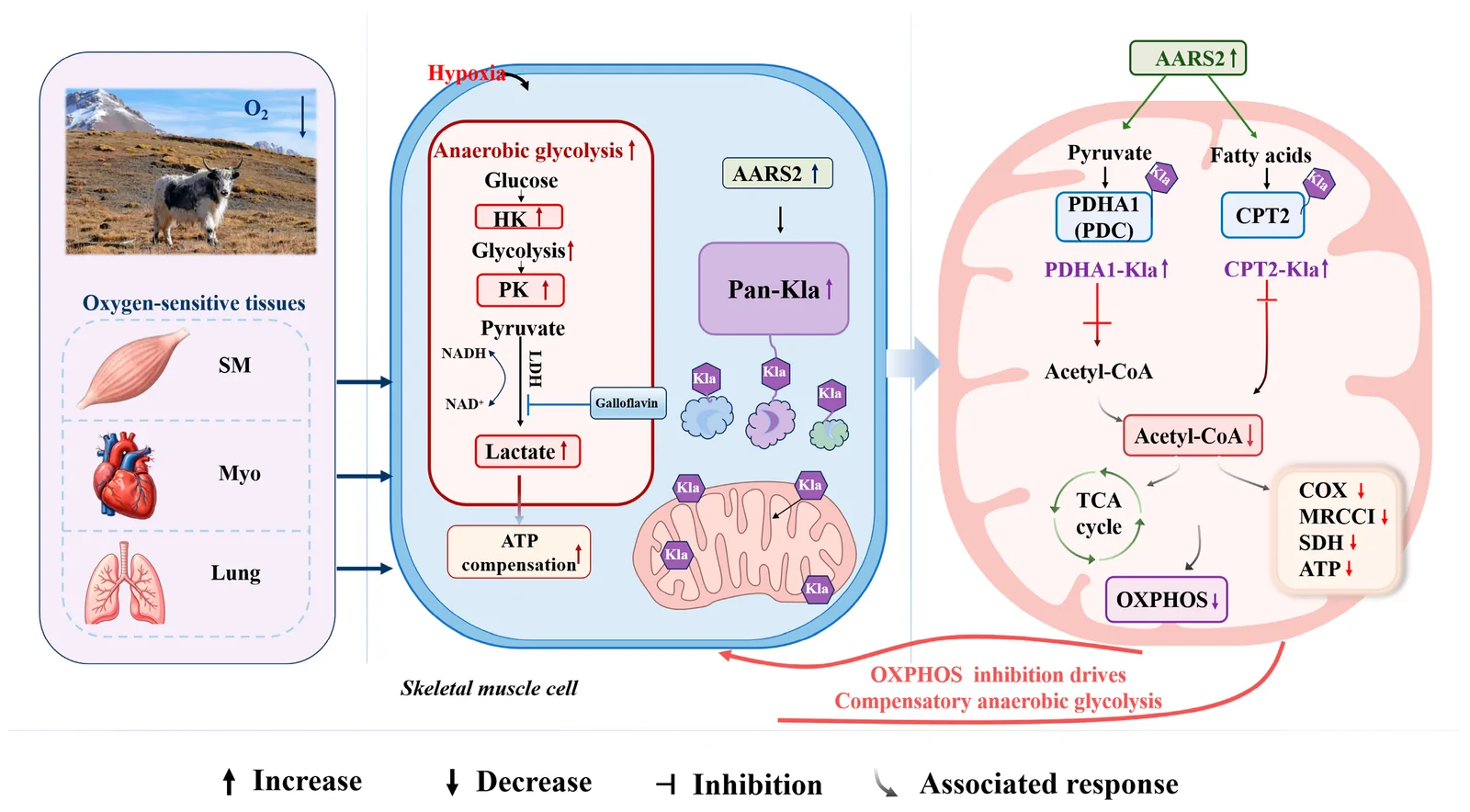
Schematic summary of the associations among hypoxia, intracellular lactate accumulation, AARS2 expression, PDHA1/CPT2 lactylation, and energy-metabolic changes in yak skeletal muscle cells. Under hypoxic conditions, intracellular lactate levels and AARS2 expression increased, whereas PDHA1 and CPT2 protein expression decreased, and both PDHA1 and CPT2 were found to undergo lactylation. These molecular changes were accompanied by increased glycolysis-related indicators and decreased mitochondrial oxidative phosphorylation-related indicators. Galloflavin treatment and AARS2 knockdown further supported associations of lactate and AARS2 with these hypoxia-associated molecular and metabolic changes. The schematic summarizes the associations observed in the present study; a direct causal relationship between AARS2, PDHA1/CPT2 lactylation, and metabolic remodeling was not established.

Lactylation is an emerging form of protein post-translational modification [23] that rapidly responds to hypoxic stress and directly regulates the activity of metabolic enzymes [24,25]. However, its role in the hypoxic response of high-altitude animals remains unclear. In this study, we systematically investigated lactylation-related changes in yak tissues across three altitude-associated sampling groups and in primary yak skeletal muscle cells under hypoxic conditions. Our findings show that hypoxia increases intracellular lactate levels and enhances global protein lactylation in yak skeletal muscle cells. Additionally, in these cells, the hypoxia-associated increase in intracellular lactate was accompanied by increased AARS2 expression and changes in indicators of glycolysis and mitochondrial oxidative phosphorylation. PDHA1 and CPT2 were found to undergo lactylation, and galloflavin treatment and AARS2 knockdown experiments further supported associations of lactate and AARS2 with these hypoxia-associated molecular and metabolic changes (Figure 9). These findings provide new insights into lactylation-related metabolic responses to hypoxia in yak skeletal muscle.

AARS2, a recently identified lysine lactyltransferase, catalyzes hypoxia-induced lactylation of mitochondrial proteins [14,26,27]. Mao et al. [14] showed that in addition to its role in mitochondrial protein translation, AARS2 directly binds to and catalyzes the lactylation of PDHA1 and CPT2, thereby limiting mitochondrial oxidative phosphorylation efficiency. Consistently, the present study showed that AARS2 expression differed among the three altitude-associated sampling groups, with generally higher levels observed in tissues from the high-altitude group. Increased AARS2 expression was also accompanied by higher global protein lactylation in skeletal muscle, supporting an association between AARS2 expression and protein lactylation [28,29] (Figure 2 and Figure 4). PDHA1 and CPT2 play important but distinct roles in mitochondrial substrate metabolism. PDHA1 is a catalytic component of the pyruvate dehydrogenase complex that links glycolysis to the tricarboxylic acid cycle, whereas CPT2 participates in mitochondrial long-chain fatty acid oxidation by converting acylcarnitines to acyl-CoAs [30,31,32,33]. In the present study, the results obtained at both the tissue and cellular levels showed that AARS2 upregulation was accompanied by significant downregulation of PDHA1 and CPT2. Moreover, siRNA-mediated knockdown of AARS2 was accompanied by increased PDHA1 and CPT2 protein levels, further supporting an inverse association between AARS2 expression and PDHA1/CPT2 protein expression in yak skeletal muscle cells (Figure 8). Taken together, the coordinated changes in AARS2, PDHA1, and CPT2 protein expression may be associated with alterations in both carbohydrate- and lipid-derived mitochondrial substrate metabolism under hypoxic conditions. However, because the catalytic activities of PDHA1 and CPT2 and cellular oxygen consumption were not directly measured in the present study, the functional consequences of these expression changes require further investigation.

Under hypoxic conditions, remodeling of energy metabolism is a key mechanism for maintaining internal homeostasis [34,35,36,37]. The present study showed that hypoxic treatment significantly increased the activities of key glycolytic enzymes (HK and PK) in yak skeletal muscle cells while decreasing those of mitochondrial oxidative phosphorylation-related enzymes (SDH, COX, and MRCCI) and reducing ATP levels, indicating a metabolic shift from oxidative phosphorylation to glycolysis (Figure 6). To further examine the association between protein lactylation and these metabolic changes, we considered the changes in PDHA1 and CPT2 together with the measured energy metabolism-related indicators. Under hypoxic conditions, PDHA1 and CPT2 expression levels decreased, and both proteins underwent lactylation (Figure 3 and Figure 7). These changes were accompanied by increased HK and PK activities and decreased activities of mitochondrial oxidative phosphorylation-related enzymes, such as SDH. PDHA1 and CPT2 are key molecules that maintain the supply of carbon substrates to mitochondria [38,39]. Under normoxic conditions, PDHA1 catalyzes the conversion of pyruvate to acetyl-CoA, which enters the TCA cycle to support oxidative phosphorylation [40]. Meanwhile, CPT2 transports acylcarnitine for conversion to acyl-CoA, thereby ensuring efficient fatty acid β-oxidation [41]. Under hypoxic conditions, however, AARS2 upregulation was accompanied by decreased PDHA1 and CPT2 protein expression, and both PDHA1 and CPT2 were found to undergo lactylation. These changes were accompanied by reduced mitochondrial oxidative phosphorylation-related indicators and increased glycolysis-related indicators under hypoxic conditions [14]. Therefore, PDHA1 and CPT2 are key molecules linking carbohydrate and lipid metabolism to mitochondrial aerobic respiration in yaks, and changes in their protein expression and lactylation may be associated with hypoxia-induced remodeling of cellular energy metabolism.

Lactate has long been regarded as a metabolic byproduct of anaerobic glycolysis [42,43,44]. However, many recent studies have shown that lactate is not merely a metabolic waste product but also an important signaling molecule that participates in the cellular hypoxic response [45,46]. Our study showed that hypoxia significantly increased lactate levels and elevated overall protein lactylation in yak skeletal muscle cells, suggesting an association between increased intracellular lactate levels and enhanced protein lactylation (Figure 4). Co-immunoprecipitation (co-IP) assays further showed that both PDHA1 and CPT2 underwent lactylation in yak skeletal muscle cells (Figure 7). Furthermore, under hypoxic conditions, AARS2 protein expression was increased, whereas PDHA1 and CPT2 protein expression was decreased. Galloflavin treatment partially attenuated these expression patterns, accompanied by decreased glycolytic enzyme activity, increased oxidative phosphorylation-related enzyme activity, and increased ATP levels. Collectively, these findings support associations between increased intracellular lactate levels and changes in AARS2 expression, PDHA1/CPT2 lactylation, and cellular energy metabolism under hypoxic conditions. This study provides a theoretical basis for further understanding the metabolic responses of yaks to hypoxia from the perspective of energy metabolism.

Given the exploratory and cross-sectional nature of the tissue analysis, with only three animals included in each altitude-associated sampling group, potential confounding effects related to geographic location, management conditions, diet, environmental factors, and genetic background cannot be fully excluded. Accordingly, the observed tissue differences should be interpreted with caution and should not be regarded as direct evidence of high-altitude adaptation. In addition, although PDHA1 and CPT2 were identified as lactylated proteins and their protein expression changed in association with AARS2 expression, the present study did not identify specific lactylation sites or directly demonstrate that AARS2 mediates the lactylation of PDHA1 and CPT2. PDHA1 and CPT2 lactylation was not assessed following AARS2 knockdown, and an AARS2 re-expression rescue experiment was not performed; therefore, nonspecific or off-target effects of the siRNA cannot be completely excluded. Furthermore, the catalytic activities of PDHA1 and CPT2 and mitochondrial respiration were not directly measured, limiting the functional interpretation of the observed changes in protein expression and metabolic indicators. Future studies incorporating mass spectrometry-based lactylation-site mapping, assessment of PDHA1/CPT2 lactylation following AARS2 knockdown, AARS2 re-expression rescue experiments, direct measurements of PDHA1/CPT2 enzymatic activities, and mitochondrial respiration analyses will be important to further validate these associations and clarify the underlying mechanisms.

## 5. Conclusions

In summary, hypoxia increased intracellular lactate levels and AARS2 expression in yak skeletal muscle cells, while PDHA1 and CPT2 protein expression decreased, and both PDHA1 and CPT2 were found to undergo lactylation. These results support an association among lactate, AARS2 expression, PDHA1/CPT2 lactylation, and hypoxia-related metabolic remodeling, and provide a theoretical basis for further understanding yaks’ metabolic responses to hypoxia.

## Figures and Tables

**Table 1:** Primer sequences used for RT-qPCR and siRNA interference.

Primer Name	Primer Sequence (5′-3′)	Product (bp)
*AARS2*-F	CATTCGAAGGTCGCCCTCA	337
*AARS2*-R	GGTATGATGGGAAAGGTCTCGG	
*PDHA1*-F	GTGCTGGTGGCATCTCGTCAT	366
*PDHA1*-R	ACCACCTCTTCGTCCTGTAAG	
*CPT2*-F	CTATGCACTACCAGGACAGCCTG	591
*CPT2*-R	AATGGAACACTTCTGGCTCC	
Si-*AARS2*-1	GCUGCUGACUCAGUGCUAUTT	486
Si-*AARS2*-2	GCUGGUAGAGCUUUGGAAUTT	732

## Data Availability

The data that support the findings of this study are available from the corresponding author upon reasonable request.

## Supplementary Materials

The following supporting information can be downloaded at: https://www.mdpi.com/article/10.3390/cells15171515/s1: Supplementary Figure S1, Effects of galloflavin on the viability of primary yak skeletal muscle cells; Supplementary Table S1, Sampling locations, environmental conditions, diet composition, housing conditions, and management practices of yaks from the three altitude-associated sampling groups; Supplementary Table S2, Exact *p* values for pairwise comparisons of *AARS2*, *CPT2*, and *PDHA1* expression among the three altitude groups in yak tissues; and Supplementary Table S3, Exact *p* values for statistical comparisons in Figure 6.

## Abbreviations

The following abbreviations are used in this manuscript:

AARS2	alanyl-transfer RNA synthetase 2
ATP	adenosine triphosphate
COX	cytochrome c oxidase
co-IP	co-immunoprecipitation
CPT1	carnitine palmitoyltransferase 1
CPT2	carnitine palmitoyltransferase 2
DMSO	dimethyl sulfoxide
ELISA	enzyme-linked immunosorbent assay
GAPDH	glyceraldehyde-3-phosphate dehydrogenase
GF	galloflavin
HK	hexokinase
IF	immunofluorescence
IP	immunoprecipitation
Kla	lysine lactylation
MRCCI	mitochondrial respiratory chain complex I
Pan-Kla	pan-lysine lactylation
PBS	phosphate-buffered saline
PDC	pyruvate dehydrogenase complex
PDHA1	pyruvate dehydrogenase E1 alpha 1
PK	pyruvate kinase
RIPA	radioimmunoprecipitation assay
SDH	succinate dehydrogenase
siRNA	small interfering RNA
TCA	tricarboxylic acid
