# Supplementary material for: Association of Hypoxia-Induced Lactate Accumulation with AARS2 Expression, PDHA1/CPT2 Lactylation, and Energy Metabolism in Yak Skeletal Muscle Cells
Source: Cells. 2026 Aug 23;15(17):1515. doi: 10.3390/cells15171515 (PMC13564726; doi:10.3390/cells15171515)
Supplement: Supplementary file 1 [file cells-15-01515-s001.zip › ARRIVE checklist(revised).pdf]

NOTE: Please save this file locally before filling in the table, DO NOT work on the file within your internet browser as changes will not be saved. Adobe Acrobat Reader (available free here) is recommended for completion.

The ARRIVE guidelines 2.0: author checklist

The ARRIVE Essential 10

These items are the basic minimum to include in a manuscript. Without this information, readers and reviewers cannot assess the reliability of the findings.

Item		Recommendation	Section/line number, or reason for not reporting
**Study design**	1	For each experiment, provide brief details of study design including:<br>a. The groups being compared, including control groups. If no control group has been used, the rationale should be stated.<br>b. The experimental unit (e.g. a single animal, litter, or cage of animals).	1a.<br>The animal-tissue component was designed as an exploratory cross-sectional comparison of yaks sampled from three geographic regions at different altitudes: low-altitude (LA) medium-altitude (MA), and high-altitude (HA) sampling groups, with three animals per group. Primary yak skeletal muscle cells were [illegible]<br>1b.<br>Each individual yak served as the experimental unit for the animal-tissue analyses. For the primary cell experiments, cells independently derived from each donor [illegible]
**Sample size**	2	a. Specify the exact number of experimental units allocated to each group, and the total number in each experiment. Also indicate the total number of animals used.<br>b. Explain how the sample size was decided. Provide details of any *a priori* sample size calculation, if done.	2a.<br>Nine animals were included in total, with three independent animals in each altitude-associated sampling group (n = 3 per group; n = 9 in total). Most primary cell experiments were performed using cells [illegible]<br>2b.<br>No a priori sample-size calculation was performed. The sample size was determined with reference to comparable published studies in this field. The limited animal sample size is explicitly acknowledged as a limitation [illegible]
**Inclusion and exclusion criteria**	3	a. Describe any criteria used for including and excluding animals (or experimental units) during the experiment, and data points during the analysis. Specify if these criteria were established *a priori*. If no criteria were set, state this explicitly.<br>b. For each experimental group, report any animals, experimental units or data points not included in the analysis and explain why. If there were no exclusions, state so.<br>c. For each analysis, report the exact value of *n* in each experimental group.	3a.<br>Healthy 12-month-old male yaks without known underlying diseases were included in the study.<br>3b.<br>No animals, experimental units, or data points were excluded from the reported analyses.<br>3c.<br>For the animal-tissue analyses, n = 3 independent animals were included in each altitude-associated sampling group. For most [illegible]
**Randomisation**	4	a. State whether randomisation was used to allocate experimental units to control and treatment groups. If done, provide the method used to generate the randomisation sequence.<br>b. Describe the strategy used to minimise potential confounders such as the order of treatments and measurements, or animal/cage location. If confounders were not controlled, state this explicitly.	4a.<br>Random allocation of animals to altitude-associated sampling groups was not applicable because the animal-tissue component was an exploratory cross-sectional study involving yaks sampled from three geographically distinct regions at different altitudes.<br>4b.<br>Because animals were not experimentally allocated to altitude groups, a randomisation sequence was not applicable. Potential confounding factors associated with geographic location, herd origin, diet, management conditions, environmental conditions, genetic background [illegible]
**Blinding**	5	Describe who was aware of the group allocation at the different stages of the experiment (during the allocation, the conduct of the experiment, the outcome assessment, and the data analysis).	No formal blinding procedure was implemented during experimental allocation, outcome assessment, or statistical analysis.
**Outcome measures**	6	a. Clearly define all outcome measures assessed (e.g. cell death, molecular markers, or behavioural changes).<br>b. For hypothesis-testing studies, specify the primary outcome measure, i.e. the outcome measure that was used to determine the sample size.	Outcome measures included AARS2, PDHA1, and CPT2 mRNA and protein expression; global protein lactylation; intracellular lactate levels; glycolysis-related and mitochondrial oxidative phosphorylation-related biochemical [illegible]<br>No single primary outcome measure was used to determine the sample size because no a priori sample-size calculation was performed. AARS2, PDHA1, and CPT2 expression, protein lactylation, and metabolic indicators were the principal outcomes [illegible]
**Statistical methods**	7	a. Provide details of the statistical methods used for each analysis, including software used.<br>b. Describe any methods used to assess whether the data met the assumptions of the statistical approach, and what was done if the assumptions were not met.	Statistical analyses were performed using GraphPad Prism 9.0. Comparisons among the three independent altitude-associated sampling groups were performed using one-way ANOVA. Comparisons between two donor-matched conditions in primary cell [illegible]<br>The assumptions underlying the statistical analyses were considered according to the experimental design and data structure. Given the small number of biological replicates, formal assessment of distributional assumptions was limited. No data [illegible]
**Experimental animals**	8	a. Provide species-appropriate details of the animals used, including species, strain and substrain, sex, age or developmental stage, and, if relevant, weight.<br>b. Provide further relevant information on the provenance of animals, health/immune status, genetic modification status, genotype, and any previous procedures.	Healthy 12-month-old male yaks (Bos grunniens) were included in the study.<br>The animals were sampled from local herds in Guide County, Haiyan County, and Golok, Qinghai Province, China, representing three geographically distinct altitude-associated sampling groups. Additional information on geographic location, environmental [illegible]
**Experimental procedures**	9	For each experimental group, including controls, describe the procedures in enough detail to allow others to replicate them, including:<br>a. What was done, how it was done and what was used.<br>b. When and how often.<br>c. Where (including detail of any acclimatisation periods).<br>d. Why (provide rationale for procedures).	9a.<br>Most primary cell experiments comprised three independent donor-derived biological replicates (n = 3), whereas the biochemical [illegible]<br>b. Cells were treated with hypoxia for 24 hours; samples were collected immediately after the animals were sacrificed<br>c. Animals were raised under standard conditions prior to sacrifice; cells underwent a 72-hour acclimation culture after thawing<br>9d.<br>The cross-sectional animal-tissue comparison was performed to characterize altitude-associated expression patterns of AARS2, PDHA1, CPT2, and protein [illegible]
**Results**	10	For each experiment conducted, including independent replications, report:<br>a. Summary/descriptive statistics for each experimental group, with a measure of variability where applicable (e.g. mean and SD, or median and range).<br>b. If applicable, the effect size with a confidence interval.	10a.<br>All quantitative data are presented as mean ± SEM. For the animal-tissue analyses, each altitude-associated sampling group contained three independent animals (n = 3 biological replicates per group). Most primary cell [illegible]<br>10b. Individual biological replicate values, mean ± SEM, and exact P values are provided. Given the exploratory nature of the study and the relatively small number of independent biological replicates, effect sizes and confidence intervals were not reported [illegible]

The Recommended Set

These items complement the Essential 10 and add important context to the study. Reporting the items in both sets represents best practice.

Item		Recommendation	Section/line number, or reason for not reporting
**Abstract**	11	Provide an accurate summary of the research objectives, animal species, strain and sex, key methods, principal findings, and study conclusions.	The study investigated yaks (Bos grunniens),
**Background**	12	a. Include sufficient scientific background to understand the rationale and context for the study, and explain the experimental approach.<br>b. Explain how the animal species and model used address the scientific objectives and, where appropriate, the relevance to human biology.	12a.<br>Hypoxia is a major environmental feature of the Qinghai–Tibet Plateau and is associated with substantial metabolic responses in high-altitude animals. Protein lactylation is an emerging post-translational modification linke<br>12b.<br>Yaks are naturally exposed to high-altitude hypoxic environments and therefore provide a relevant animal model for investigating hypoxia-related metabolic responses. The combination of yak tissue samples and
**Objectives**	13	Clearly describe the research question, research objectives and, where appropriate, specific hypotheses being tested.	The study investigated whether AARS2 expres
**Ethical statement**	14	Provide the name of the ethical review committee or equivalent that has approved the use of animals in this study, and any relevant licence or protocol numbers (if applicable). If ethical approval was not sought or granted, provide a justification.	14.<br>All animal handling and sampling procedures were reviewed and approved by the Animal Welfare and Research Ethics Committee of Qinghai University (IACUC Approval No. SL-2023005, approved on 15 March 2023). All procedures were conducted in accordance with the 3Rs principles for animal experimentation.
**Housing and husbandry**	15	Provide details of housing and husbandry conditions, including any environmental enrichment.	Yaks in the three altitude-associated sampling
**Animal care and monitoring**	16	a. Describe any interventions or steps taken in the experimental protocols to reduce pain, suffering and distress.<br>b. Report any expected or unexpected adverse events.<br>c. Describe the humane endpoints established for the study, the signs that were monitored and the frequency of monitoring. If the study did not have humane endpoints, state this.	16a.<br>The animal component of the study was an exploratory cross-sectional tissue-sampling study and did not involve experimentally induced disease or prolonged experimental treatment of live animals. Animals were<br>16b.<br>No study-specific live-animal intervention was performed before slaughter; therefore, study-related adverse events associated with an experimental treatment were not applicable to the animal-tissue component.<br>16c.<br>Study-specific humane endpoints were not established because the animal component did not involve prolonged experimental treatment or disease induction in live animals; tissue samples were collected immediately
**Interpretation/ scientific implications**	17	a. Interpret the results, taking into account the study objectives and hypotheses, current theory and other relevant studies in the literature.<br>b. Comment on the study limitations including potential sources of bias, limitations of the animal model, and imprecision associated with the results.	17a.<br>The results are interpreted as showing associations among hypoxia, intracellular lactate accumulation, AARS2 expression, PDHA1/CPT2 lactylation, and changes in energy metabolism. Hypoxia increased<br>17b.<br>The animal-tissue component was exploratory and cross-sectional, with only three animals included in each altitude-associated sampling group. Because the three groups originated from different geographic regions, potential
**Generalisability/ translation**	18	Comment on whether, and how, the findings of this study are likely to generalise to other species or experimental conditions, including any relevance to human biology (where appropriate).	18.<br>The findings are based on yak tissues and primary yak skeletal muscle cells and therefore should be generalized to other species or experimental conditions with caution. The animal-tissue observations were obtained from an exploratory cross-sectional comparison of three geographically distinct altitude-associated sampling groups, while the
**Protocol registration**	19	Provide a statement indicating whether a protocol (including the research question, key design features, and analysis plan) was prepared before the study, and if and where this protocol was registered.	A study protocol and analysis plan were prepared before the experiments; however, the protocol was not preregistered in a public repository.
**Data access**	20	Provide a statement describing if and where study data are available.	20.Complete numerical source data underlyin
**Declaration of interests**	21	a. Declare any potential conflicts of interest, including financial and non-financial. If none exist, this should be stated.<br>b. List all funding sources (including grant identifier) and the role of the funder(s) in the design, analysis and reporting of the study.	a. All authors of this manuscript declare no competing financial interests or personal relationships that could potentially influence the reported research outcomes.<br>21b. This research was jointly supported by the Youth Project of the Basic Research Program of Qinghai Provincial Department of Science and Technology (Grant No. 2025-ZJ-980Q) and the National Natural Science Foundation of China (Grant No.
