# Supplementary material for: Association of Hypoxia-Induced Lactate Accumulation with AARS2 Expression, PDHA1/CPT2 Lactylation, and Energy Metabolism in Yak Skeletal Muscle Cells
Source: Cells. 2026 Aug 23;15(17):1515. doi: 10.3390/cells15171515 (PMC13564726; doi:10.3390/cells15171515)
Supplement: Supplementary file 1 [file cells-15-01515-s001.zip › Supplementary Figure S1.pdf]

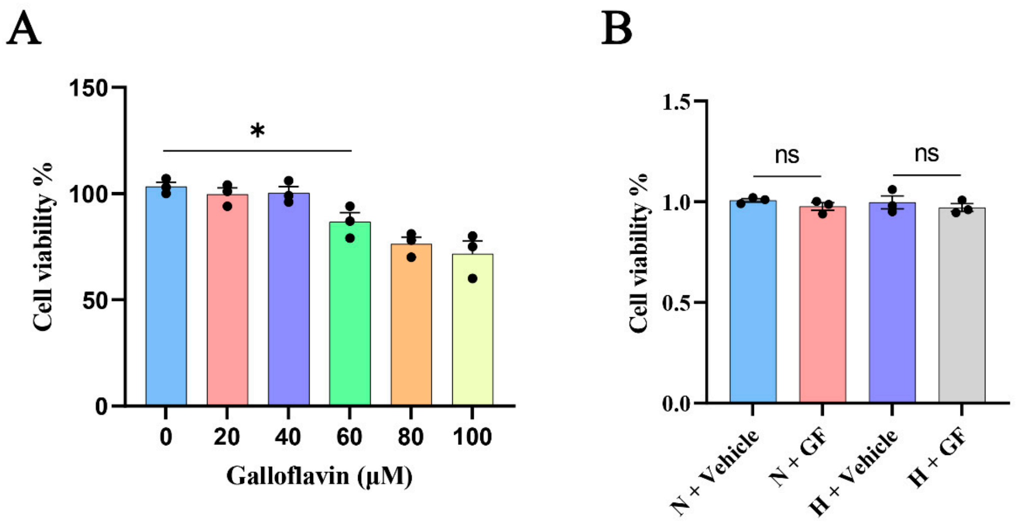

Supplementary Figure S1. Effects of galloflavin on the viability of primary yak skeletal muscle cells. (A) Cell viability was assessed using the CCK-8 assay after treatment with the indicated concentrations of galloflavin (0, 20, 40, 60, 80, and 100 μM) for 24 h under normoxic conditions. The 0 μM group received DMSO alone and served as the vehicle control, and the final DMSO concentration was kept consistent among all groups. (B) Cell viability was assessed after treatment with DMSO vehicle or 50 μM galloflavin for 24 h under normoxic or hypoxic conditions. Data are presented as mean ± SEM from three independent biological replicates (n = 3), with individual data points representing independent biological replicates. Statistical significance in (A) was assessed by one-way ANOVA followed by Dunnett's multiple-comparisons t est u sing t he D MSO v ehicle g roup a s t he c ontrol, w hereas comparisons in (B) were performed between DMSO vehicle- and galloflavin-treated cells within each oxygen condition. $P < 0.05$; ns, not significant.
